# Early Pacemaker Dependency After Heart Transplantation Is Associated with Permanent Pacemaker Implantation, Graft Failure and Mortality

**DOI:** 10.3390/jcdd11120394

**Published:** 2024-12-08

**Authors:** Fabrice F. Darche, Karsten M. Heil, Rasmus Rivinius, Matthias Helmschrott, Philipp Ehlermann, Norbert Frey, Ann-Kathrin Rahm

**Affiliations:** 1 Department of Cardiology, Angiology and Pneumology, Heidelberg University Hospital, 69120 Heidelberg, Germany; 2 Heidelberg Center for Heart Rhythm Disorders (HCR), Heidelberg University Hospital, 69120 Heidelberg, Germany

**Keywords:** atrioventricular block, bradycardia, cardiac arrythmias, heart transplantation, pacemaker, survival

## Abstract

Aims: Patients after heart transplantation (HTX) often experience post-transplant bradycardia, but little is known about the outcomes of early pacemaker dependency after HTX. We compared post-transplant mortality, graft failure, and the requirement for the permanent pacemaker implantation of patients with and without early pacemaker dependency after HTX. Methods: We screened all adult patients for early pacemaker dependency after HTX (defined as immediately after surgery) who underwent HTX at Heidelberg Heart Center between 1989 and 2022. Patients were stratified by diagnosis and type of early pacemaker dependency after HTX (sinoatrial or atrioventricular conduction disturbance). Results: A total of 127 of 699 HTX recipients (18.2%) had early pacemaker dependency after HTX, including 52 patients with sinoatrial conduction disturbances (40.9%) and 75 patients with atrioventricular conduction disturbances (59.1%). Patients with early pacemaker dependency after HTX showed both increased 1-year overall mortality after HTX (55.9% vs. 15.2%, *p* < 0.001) and higher mortality due to graft failure (25.2% vs. 4.2%, *p* < 0.001). Multivariate analysis revealed early pacemaker dependency after HTX (HR: 5.226, 95% CI: 3.738–7.304, *p* < 0.001) as an independent risk factor for 1-year mortality after HTX. Patients with early pacemaker dependency after HTX had a higher rate of 30-day (7.1% vs. 0.4%, *p* < 0.001) and 1-year (11.8% vs. 0.5%, *p* < 0.001) permanent pacemaker implantation after HTX compared to patients without early pacemaker dependency after HTX. Conclusions: Patients with early pacemaker dependency after HTX had a significantly higher rate of post-transplant mortality, graft failure, and the requirement for permanent pacemaker implantation.

## 1. Introduction

Heart rhythm disorders are common after heart transplantation (HTX) and may pose a relevant threat to HTX recipients [1,2,3]. Besides tachycardic heart rhythm disorders after HTX including atrial fibrillation, atrial flutter, and sinus tachycardia, bradycardic heart rhythm disorders commonly found after HTX are sinoatrial and atrioventricular conduction disturbances [4,5,6,7,8,9,10]. Although post-transplant bradycardias are often temporary and can spontaneously recover, the implantation of a permanent pacemaker (PPM) may be needed in some HTX recipients with symptomatic or persistent post-transplant bradycardias [10,11,12]. Previous publications have reported post-transplant PPM implantation rates between 3.5% and 20.5% [13,14,15] including a large analysis of the United Network for Organ Sharing (UNOS) database, showing that about 10% of all HTX recipients have received a post-transplant PPM implantation [14].

Several risk factors for bradycardic heart rhythm disorders after HTX have been described including graft failure, acute rejection, older donor age, biatrial anastomosis, prolonged ischemic time, the use of negative chronotropic drugs, and chronic kidney disease with electrolyte imbalance [10,13,16,17,18,19]. The use of biatrial anastomosis has especially been associated with sinus node dysfunction and higher rates of PPM implantation after HTX as it comprises a long circular suture between the right recipient atrium and the right donor atrium, resulting in an elongated new right atrium with a distorted anatomy, an abundance of scar tissue, and possible sinus node injury [9,10,11,20,21,22,23,24].

Early bradycardic heart rhythm disorders immediately after HTX may be transitorily treated with the help of temporary pacemaker systems, which are routinely placed during HTX surgery but may also require PPM implantation if they persist [10,13,17,18,19]. However, little is known about the outcomes of bradycardias with early pacemaker dependency immediately after HTX. Given the limited available published knowledge in this area, we decided to investigate the post-transplant effects of early pacemaker dependency immediately after HTX in a large cohort of HTX recipients, focusing on mortality, causes of death, and the requirement for PPM implantation after HTX.

## 2. Patients and Methods

### 2.1. Patients

We performed this study in accordance with the ethical standards of the Declaration of Helsinki. The institutional review board (IRB) of Heidelberg University, Heidelberg, Germany, gave its approval (ethics approval number: S-286/2015, Version 1.2, 28 July 2020. We obtained written informed consent from patients for their inclusion in the Heidelberg HTX Registry and the clinical and scientific use of their data. The ethics approval did not require additional consent for this observational study as only routine clinical data were utilized [5,6,7,8,9,10].

We screened the medical data of all adult patients (≥18 years) for early post-transplant pacemaker dependency who underwent HTX at Heidelberg Heart Center, Heidelberg, Germany, between 1989 and 2022. Patients who had undergone repeat HTXs were excluded. Pacemaker dependency was defined as the presence of pacemaker activity when the pacing rate was programmed at 50 beats/min [25,26]. Patients with early pacemaker dependency after HTX were further stratified into patients with sinoatrial conduction disturbance after HTX and patients with atrioventricular conduction disturbance after HTX. Our stratification was based on the findings in the electrocardiogram and reports that were immediately performed after surgery. The interpretation of the electrocardiogram was carried out in accordance with the recommendations for the standardization and interpretation [27,28,29].

### 2.2. Follow-Up

A follow-up of HTX recipients was performed in accordance with Heidelberg Heart Center’s routine clinical protocol. Patients received a temporary pacemaker system consisting of an external pacing box and two epicardial pacing leads that were placed on the right atrium and ventricle during HTX surgery. The epicardial pacing leads remained in situ for about 10 days after HTX. During the initial hospital stay, 12-lead electrocardiography (ECG) was performed in routine follow-up intervals. Additionally, resting ECG was performed in cases of suspected arrhythmia. Prior to discharge, patients routinely had a 24-h Holter recording performed [5,6,7,8,9,10].

After hospital discharge following HTX, patients were seen monthly as outpatients in the HTX clinic during the first six post-transplant months, then bimonthly until the end of the first year after HTX, and approximately three to four times per year thereafter (with additional visits as clinically needed) [5,6,7,8,9,10].

Post-transplant routine follow-up included medical history, physical examination, systolic and diastolic blood pressure measurement, blood and laboratory tests including immunosuppressive drug monitoring, resting 12-lead ECG, echocardiography, endomyocardial biopsy, annual chest X-rays as well as annual 24-h Holter monitoring. We were able to obtain complete follow-up data after HTX from all patients as no patient was lost to follow-up [5,6,7,8,9,10].

### 2.3. Post-Transplant Medications

Medications after HTX including immunosuppressive drugs were administered as per the Heidelberg Heart Center’s standard of care. Perioperatively, patients received anti-thymocyte globulin-based immunosuppression induction therapy. Cyclosporine A and azathioprine were used as the initial immunosuppressive regimen prior to 2001. From 2001, mycophenolic acid replaced azathioprine subsequently and tacrolimus replaced cyclosporine A from 2006 onward. Steroids were gradually tapered and eventually discontinued six months after HTX (unless clinically required for a longer period) [5,6,7,8,9,10].

### 2.4. Statistical Analysis

Data were analyzed using MedCalc (Version 22.032, MedCalc Software Ltd., Ostend, Belgium) and shown as the mean ± standard deviation (SD) or as a count (*n*) with a percentage (%). For measures of association, the mean difference (MD) with a 95% confidence interval (CI) was applied. Depending on the variable type and question, we used Student’s *t*-test, the Mann–Whitney U-test, an analysis of variance (ANOVA), the Kruskal–Wallis test, the chi-squared test, or Fisher’s exact test, as appropriate. The Kaplan–Meier estimator using the log-rank test was applied to graphically compare survival after HTX between patients with and without early pacemaker dependency after HTX. A *p*-value of <0.050 was considered statistically significant [5,6,7,8,9,10].

The primary outcome of this study was 30-day and 1-year post-transplant mortality between patients with and without early pacemaker dependency after HTX, as well as between patients with sinoatrial conduction disturbance after HTX and patients with atrioventricular conduction disturbance after HTX. Causes of death were categorized into the following groups: graft failure, acute rejection, infection/sepsis, malignancy, and thromboembolic event/bleeding. An analysis of post-transplant mortality further included a multivariate analysis for 1-year mortality after HTX (the Cox regression model) with the following eight clinically relevant parameters: recipient age, recipient body mass index (BMI), recipient estimated glomerular filtration rate (eGFR), donor age, donor BMI, biatrial anastomoses, total ischemic time, and early pacemaker dependency after HTX. We did not include additional variables in this multivariate analysis for 1-year mortality after HTX to avoid biased regression coefficients and to ensure a stable number of events (deceased patients) per analyzed variable [5,6,7,8,9,10].

Secondary outcomes included an analysis of 30-day and 1-year permanent pacemaker implantation after HTX between patients with and without early pacemaker dependency after HTX. Additionally, we performed univariate analyses to search for intergroup differences between patients with and without early pacemaker dependency after HTX, as well as between patients with early sinoatrial conduction disturbance after HTX and patients with early atrioventricular conduction disturbance after HTX. The analyzed variables included recipient data, recipient previous open-heart surgery, recipient principal diagnosis for HTX, donor data, transplant sex mismatch, perioperative data, immunosuppressive drug therapy, and post-transplant concomitant medications [5,6,7,8,9,10].

With respect to the length of the study period of more than 30 years, we performed a sensitivity analysis to test the robustness of our results and to examine a possible era effect using a subgroup of patients with tacrolimus and mycophenolic acid as the immunosuppressive drug regimen was changed from 2006 onward [5,6,7,8,9,10].

## 3. Results

### 3.1. Post-Transplant Demographics

We could include 699 HTX recipients after applying the exclusion criteria. A total of 127 of 699 HTX recipients (18.2%) had early pacemaker dependency after HTX, including 52 patients with early sinoatrial conduction disturbances (52 of 127 [40.9%]) and 75 patients with early atrioventricular conduction disturbances (75 of 127 [59.1%]).

Patients with early pacemaker dependency after HTX had a higher donor age (45.0 ± 13.0 years vs. 40.9 ± 13.6 years, MD: 4.1 years, CI: 1.6–6.6 years, *p* = 0.002), a higher rate of biatrial anastomosis (35.4% vs. 21.0%, MD: 14.4%, CI: 5.4–23.4%, *p* = 0.001), and a higher fraction of recipient chronic kidney disease (66.9% vs. 54.7%, MD: 12.2%, CI: 3.1–21.3%, *p* = 0.012) along with a lower recipient eGFR (55.9 ± 20.8 mL/min/1.73 m^2^ vs. 61.4 ± 22.0 mL/min/1.73 m^2^, MD: 5.5 mL/min/1.73 m^2^, CI: 1.5–9.5 mL/min/1.73 m^2^, *p* = 0.008) compared to patients without early pacemaker dependency after HTX. No other significant differences between both groups were found concerning the other parameters. Demographics stratified by early pacemaker dependency after HTX are shown in Table 1.

A further subgroup analysis of patients with early pacemaker dependency after HTX showed that patients with early sinoatrial conduction disturbance more frequently received biatrial anastomosis in comparison to patients with early atrioventricular conduction disturbance after HTX (59.6% vs. 18.7%, MD: 40.9%, CI: 24.9–56.9%, *p* < 0.001), whereas there were no statistically significant differences in terms of the remaining parameters. Demographics stratified by early conduction disturbance after HTX are presented in Table 2.

### 3.2. Post-Transplant Medications

As for the immunosuppressive drug therapy, we could neither detect statistically significant differences between patients with and without early pacemaker dependency after HTX nor between patients with early sinoatrial conduction disturbance after HTX or patients with atrioventricular conduction disturbance after HTX in relation to the use of cyclosporine A, tacrolimus, everolimus, azathioprine, mycophenolic acid, or steroids (all *p* ≥ 0.050). Post-transplant medications stratified by early pacemaker dependency after HTX are shown in Table 3.

Likewise, we observed no statistically significant differences between patients with and without early pacemaker dependency after HTX or between patients with early sinoatrial or atrioventricular conduction disturbance after HTX regarding the administration of acetylsalicylic acid, beta blockers, ivabradine, calcium channel blockers, angiotensin-converting-enzyme inhibitors/angiotensin II receptor blockers, diuretics, statins, or gastric protection drugs (all *p* ≥ 0.050). Post-transplant medications stratified by early conduction disturbance after HTX are listed in Table 4.

### 3.3. Post-Transplant Mortality and Survival

**Figure 1:**
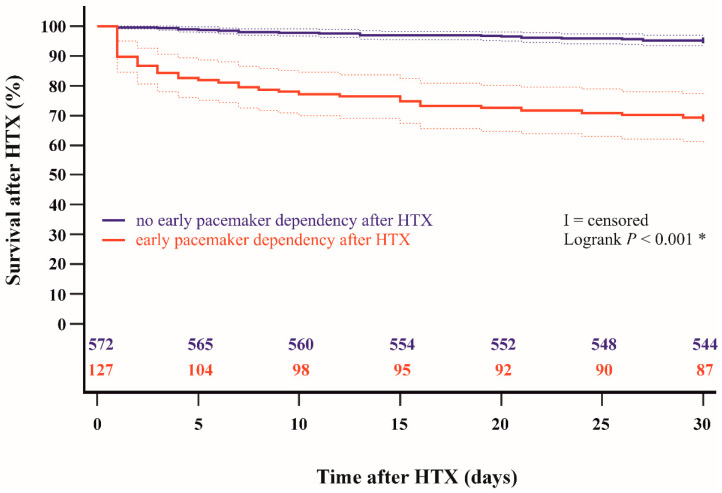
Thirty-day survival after HTX between patients with and without early pacemaker dependency after HTX (Kaplan–Meier estimator). Patients with early pacemaker dependency after HTX had a significantly lower 30-day post-transplant survival than patients without early pacemaker dependency after HTX (*p* < 0.001). HTX = heart transplantation; * = statistically significant (*p* < 0.050).

**Figure 2:**
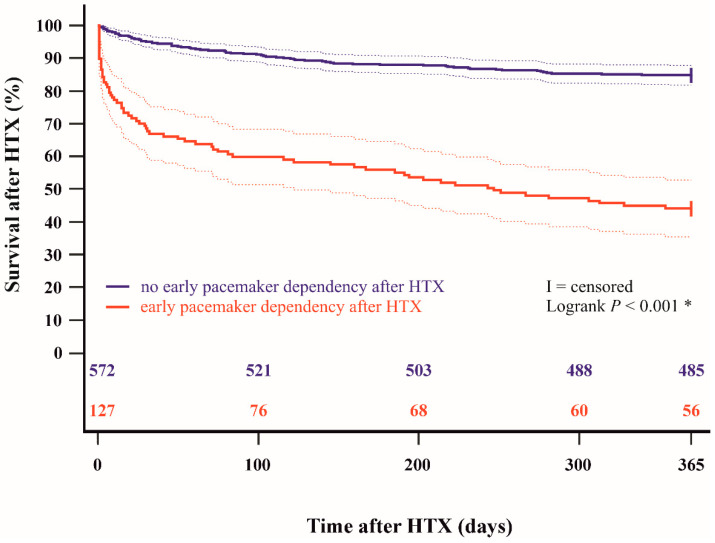
One-year survival after HTX between patients with and without early pacemaker dependency after HTX (Kaplan–Meier estimator). Patients with early pacemaker dependency after HTX had a significantly lower 1-year post-transplant survival (*p* < 0.001). HTX = heart transplantation; * = statistically significant (*p* < 0.050).

Kaplan–Meier survival analysis showed a significantly lower 30-day post-transplant survival (*p* < 0.001) and 1-year post-transplant survival (*p* < 0.001) in patients with early pacemaker dependency after HTX in comparison to patients without early pacemaker dependency after HTX. The Kaplan–Meier estimators are displayed in Figure 1 and Figure 2.

When examining causes of death within 30 days after HTX, significantly more patients with early pacemaker dependency after HTX died from graft failure (22.8% vs. 2.4%, MD: 20.4%, CI: 13.0–27.8%, *p* < 0.001), thromboembolic event/bleeding (3.2% vs. 0.7%, MD: 2.5%, CI: 0.6–5.6%, *p* = 0.019), and acute rejection (1.6% vs. 0.2%, MD: 1.4%, CI: 0.2–2.6%, *p* = 0.029). Table 5 presents the causes of death within 30 days after HTX.

Similar results were seen for causes of death within one year after HTX. Patients with early pacemaker dependency after HTX died significantly more often from graft failure (25.2% vs. 4.2%, MD: 21.0%, CI: 13.3–28.7%, *p* < 0.001), thromboembolic event/bleeding (5.5% vs. 1.2%, MD: 4.3%, CI: 0.2–8.4%, *p* = 0.002), and infection/sepsis (22.8% vs. 8.9%, MD: 13.9%, CI: 6.2–21.6%, *p* < 0.001). Causes of death within 1 year after HTX are shown in Table 6. There was no significant difference between patients with early sinoatrial or atrioventricular conduction disturbance after HTX in terms of 30-day post-transplant survival, 1-year post-transplant survival, or causes of death (all *p* ≥ 0.050).

Multivariate analysis for 1-year mortality after HTX showed that patients with early pacemaker dependency after HTX had a more than five-fold increased risk of death within 1 year after HTX (HR: 5.226, 95% CI: 3.738–7.304, *p* < 0.001), whereas the other seven included variables (recipient age, recipient body mass index, recipient estimated glomerular filtration rate, donor age, donor body mass index, biatrial anastomosis, and total ischemic time) showed no statistically significant effect on 1-year post-transplant mortality. The multivariate analysis for 1-year mortality after HTX can be found in Table 7.

### 3.4. Post-Transplant Permanent Pacemaker Implantation

Patients with early pacemaker dependency after HTX had a significantly higher rate of PPM implantation within 30 days after HTX (7.1% vs. 0.4%, MD: 6.7%, CI: 2.2–11.2%, *p* < 0.001) and within 1 year after HTX (11.8% vs. 0.5%, MD: 11.3%, CI: 5.7–16.9%, *p* < 0.001). There was no significant difference between patients with early sinoatrial or atrioventricular conduction disturbance after HTX regarding 30-day or 1-year PPM implantation after HTX (all *p* ≥ 0.050). The results for permanent pacemaker implantation after HTX are shown in Table 8.

All patients with early pacemaker dependency after HTX and PPM implantation survived within 30 days after HTX, while patients with early pacemaker dependency after HTX but without PPM implantation had a significantly higher 30-day mortality after HTX (35.7% vs. 0.0%, MD: 35.7%, CI: 26.8–44.6%, *p* = 0.005). When looking at mortality between 31 days and 365 days after HTX, 8 of 15 (53.3%) patients with early pacemaker dependency after HTX and PPM implantation deceased. Regarding the causes of death, seven of the patients died from infection/sepsis (87.5%) and one patient from graft failure (12.5%). In terms of 1-year mortality after HTX, there was no statistically significant difference between patients with early pacemaker dependency after HTX and PPM implantation in comparison to patients with early pacemaker dependency after HTX but without PPM implantation (53.3% vs. 56.3%, MD: 3.0%, CI: −23.9–29.9%, *p* = 0.831). The results for one-year mortality after HTX in patients with early pacemaker dependency after HTX stratified by PPM within one year after HTX are shown in Table 9.

### 3.5. Sensitivity Analysis

Due to the extended study period, we examined the potential for an era effect by conducting a sensitivity analysis on a subgroup of patients who received tacrolimus and mycophenolic acid as their immunosuppressive drug therapy (350 of 699 HTX recipients [50.1%]). This analysis showed comparable results, supporting the robustness of our findings and reducing the likelihood of a possible era effect. In addition, we analyzed 1-year mortality after HTX stratified by HTX era. This analysis showed a significantly higher 1-year mortality of patients with early pacemaker dependency after HTX during all HTX eras. The results for one-year mortality after HTX stratified by HTX era are provided in Table 10.

## 4. Discussion

### 4.1. Frequency and Risk Factors of Early Pacemaker Dependency After HTX

Bradycardic heart rhythm disorders after HTX are frequent and may require post-transplant pacing in HTX recipients [1,2,3,4,5,6,7,8,9,10,11,12]. However, published data concerning the percentage of early post-transplant pacing vary. Bacal and colleagues [13] reported early post-transplant pacing in 10 of 114 HTX recipients (8.8%), while Jacquet and colleagues [3] reported early post-transplant pacing in 9 of 25 HTX recipients (36.0%). In our study, we observed a total of 127 of 699 HTX recipients (18.2%) with early pacemaker dependency after HTX. Concerning the type of bradycardia requiring early pacemaker dependency after HTX, we found 52 of 127 patients with sinoatrial conduction disturbances (40.9%) and 75 of 127 patients with atrioventricular conduction disturbances (59.1%).

In terms of risk factors of early pacemaker dependency after HTX, several parameters have previously been discussed [9,10,11,12,13,14,15,16,17,18,19,20,21,22]. In our study, patients with early pacemaker dependency after HTX had a higher donor age (*p* = 0.002) and more often received biatrial anastomosis (*p* = 0.001). These findings align with those of a large multi-center study by Cantillon and colleagues [14], which reported that a higher donor age and biatrial anastomosis were associated with an increased need for a postoperative pacemaker requirement. At Heidelberg Heart Center, both surgical techniques, biatrial and bicaval anastomosis, are used. However, the decision of which surgical technique is performed is not preselected. Factors influencing the choice of HTX technique are the surgeon’s preference, anatomical characteristics, and previous open-heart surgery [9,10]. In order to evaluate the role of surgical technique (biatrial or bicaval HTX) in early pacemaker dependency after HTX, we compared the surgical techniques of patients with or without early pacemaker dependency after HTX. Patients with early pacemaker dependency after HTX had a significantly higher percentage of biatrial HTX (35.4% vs. 21.0%, *p* < 0.001), while patients without early pacemaker dependency after HTX had a significantly higher rate of bicaval HTX (79.0% vs. 64.6%, *p* < 0.001). However, multivariate analysis for 1-year mortality after HTX showed no statistically significant impact of biatrial anastomosis on 1-year post-transplant mortality (HR: 0.775, 95% CI: 0.505–1.188, *p* = 0.242), whereas early pacemaker dependency after HTX was an independent risk factor for 1-year mortality after HTX (HR: 5.226, 95% CI: 3.738–7.304, *p* < 0.001), indicating that patients with early pacemaker dependency after HTX had a more than five-fold increased risk of death within 1 year after HTX independently of the underlying surgical technique. Likewise, Dell’Aquila and colleagues [23] found no significant effect of surgical technique on mortality after HTX.

A common complication of HTX recipients with pre-existing chronic kidney disease is the further deterioration of renal function after HTX, which may lead to hyperkalemia and bradycardia, requiring pacing [10,30,31,32,33,34]. We also observed a higher percentage of chronic kidney disease in HTX recipients with early pacemaker dependency after HTX (*p* = 0.012). Additionally, prolonged ischemic time has been linked to post-transplant bradyarrhythmias, which may result from perioperative hypoxia-induced damage of the sinoatrial node and the cardiac conduction system [3,10,11,18]. However, we could not find a statistically significant difference in total, cold, or warm ischemic time between patients with or without early pacemaker dependency after HTX in our study. Similar results were observed by Wellmann and colleagues [35].

Another controversially discussed possible risk factor for early post-transplant pacing and the need for PPM implantation after HTX is the pre-transplant use of amiodarone [13,15,36,37,38,39]. Chelimsky-Fallick and colleagues [36] as well as Macdonald and colleagues [37] reported a higher need for temporary pacing after HTX due to bradycardia in patients with the pre-transplant use of amiodarone but found no significant difference in PPM implantation after HTX. In contrast, we could not observe a significant difference between patients with or without the pre-transplant use of amiodarone prior to HTX concerning post-transplant bradycardia or PPM implantation after HTX, as discussed in a previously published article [38]. This is in line with reports by Zieroth and colleagues [15] as well as by Woo and colleagues [39] who also could not find a statistically significant association between relevant bradycardia requiring PPM after HTX and the pre-transplant use of amiodarone prior to HTX.

### 4.2. Clinical Outcomes and Management of Early Pacemaker Dependency After HTX

After successful HTX, the cardiac graft is normally capable of generating a stable heart rhythm and starts beating spontaneously once cardiovascular circulation is re-established. However, an electric shock is sometimes required to induce the cardiac graft to start beating. If bradyarrhythmias after HTX should persist, reversible causes such as acute graft rejection or negative chronotropic drugs need to be addressed, followed by a prescription of positive chronotropic medications and temporary pacing, leaving PPM implantation after HTX as the final option [10,13,16,17,18,19].

At Heidelberg Heart Center, patients routinely receive a temporary epicardial lead pacing system during surgery, which usually remains in situ for up to 10 days after HTX [10]. With the help of temporary pacing, intermittent bradyarrhythmias after HTX can be safely bridged, reducing the need for PPM implantation [10,13,17,19]. In addition, the amount of positive inotropic agents such as catecholamines may be decreased given a stabilized heart rate. However, temporary pacing comes at a price such as an elevated risk for infection, lead dislodgement, inadequate pacing, and the fact that it may not be possible in all patients [10,13].

We observed no statistically significant differences in the use of beta blockers, calcium channel blockers or ivabradine between patients with or without early pacemaker dependency after HTX in this study. As a sufficient immunosuppressive drug therapy is needed to reduce the risk for acute rejection in HTX recipients [40], we also compared the immunosuppressive drug regimen of patients with or without early pacemaker dependency after HTX, which yielded no statistically significant difference regarding the use of cyclosporine A, tacrolimus, everolimus, azathioprine, mycophenolic acid, or steroids (all *p* ≥ 0.050).

Importantly, in our study, patients with early pacemaker dependency after HTX showed a significantly higher 30-day mortality after HTX due to acute rejection (*p* = 0.029) and graft failure (*p* < 0.001), along with a significantly higher 30-day all-cause mortality after HTX (*p* < 0.001), indicating a poor prognosis in patients with early pacemaker dependency after HTX. This hypothesis is supported by the fact that patients with early pacemaker dependency after HTX had a significantly higher rate of PPM implantation within 30 days after HTX (*p* < 0.001) and within 1 year after HTX (*p* < 0.001).

Although PPM implantation after HTX has generally not been considered to be associated with higher mortality after HTX [11,12,15,18,30,35], early pacemaker dependency after HTX and early PPM implantation after HTX may impact post-transplant survival [10]. In a previously published article [10], we showed that the 5-year post-transplant survival of patients with early PPM implantation after HTX was significantly lower compared to patients with late PPM implantation or no PPM implantation after HTX (*p* < 0.01). Of particular interest is the fact that this study showed no significant difference in 5-year post-transplant survival (*p* = 0.85) between patients who received PPM implantation after HTX and those who did not [10].

In contrast, Roest and colleagues [41] reported a diminished post-transplant survival in patients with late PPM implantation after HTX, while early PPM implantation after HTX did not compromise post-transplant survival. Differing from these results, Jones and colleagues [20] found no statistically significant difference in post-transplant survival between patients with early or late PPM implantation after HTX or between patients with late PPM implantation or no PPM implantation after HTX. Another study by Cantillon and colleagues [14] even published a superior post-transplant survival in patients with PPM implantation after HTX.

In terms of PPM implantation after HTX and its impact on post-transplant survival, variations between study results should be interpreted with care given the differences in study population, study size, the ratio of early vs. late PPM implantation after HTX, the definition of early PPM implantation after HTX, and the location of study [10,14,20,41].

In the current study, we observed that all patients with early pacemaker dependency after HTX and PPM implantation survived within 30 days after HTX, while more than one-third of patients with early pacemaker dependency after HTX but without PPM implantation died within 30 days after HTX, indicating that PPM implantation could be beneficial. However, when looking at post-transplant mortality between 31 days and 365 days after HTX, more than half of the patients with early pacemaker dependency after HTX and PPM implantation deceased. Regarding the causes of death, most of these patients died from infection/sepsis (87.5%) and only a minority from graft failure (12.5%). Interestingly, there was no statistically significant difference between patients with early pacemaker dependency after HTX and PPM implantation in comparison to patients with early pacemaker dependency after HTX but without PPM implantation concerning 1-year mortality after HTX (*p* = 0.831).

This could mean that post-transplant bradycardia is a strong marker of early graft failure. Some of its symptoms (bradycardia/early pacemaker dependency) might be temporarily bridged by PPM implantation but not the underlying cause of graft failure. This hypothesis is supported by the fact that most of the excess deaths in the early pacemaker dependency group within the first 30 days after HTX were due to acute rejection and graft failure, whereas between 30 days and 365 days after HTX, the cause was infection/sepsis. Although these findings may seem contradictory, it is possible that the infection/sepsis was secondary to the need for higher doses of immunosuppressive drugs or even due to a change in therapeutic strategy to treat other non-fatal rejection episodes in the early pacemaker dependency group. Therefore, our data suggest that early pacemaker dependency after HTX is a strong marker of early graft failure; however, further studies, preferably large multi-center trials, are necessary to explore this matter in detail.

### 4.3. Study Limitations

Our results were derived from a large single-center registry (Heidelberg HTX Registry). Given the known limitations of this study design, our findings should be interpreted carefully and within the context of the existing literature. Nonetheless, our analysis is the most extensive study currently available regarding the effects of early pacemaker dependency after HTX. We were able to use highly detailed data of 699 HTX recipients, as our patients received standardized treatment and follow-up, reducing the likelihood of selection bias and potential confounders [5,6,7,8,9,10].

We decided to include patients who received HTX at Heidelberg Heart Center between 1989 and 2022 to recruit a large sample size for an accurate analysis. Due to the long study period, a possible era effect due to changes in surgical and medical care may have influenced our results. We therefore investigated a possible era effect by performing a sensitive analysis using a subgroup of HTX recipients with tacrolimus and mycophenolic acid as the immunosuppressive drug regimen was changed from 2006 onward. This analysis showed similar results, supporting the robustness of our findings [5,6,7,8,9,10].

One of the study’s limitations is that the decision to place a pacemaker sometimes depends on a subjective evaluation, especially for sinus bradycardia. The threshold between what is acceptable and what requires a pacemaker may vary from one operator to another.

Importantly, our findings should be regarded as hypothesis-generating, especially with respect to survival after HTX as multiple factors may influence post-transplant survival. Our results can thus neither proof or disproof a causal relationship between early pacemaker dependency after HTX and increased post-transplant mortality but merely indicate an association. Consequently, to validate our findings, further research is needed, ideally through large multicenter trials [5,6,7,8,9,10].

## 5. Conclusions

Post-transplant bradycardias may pose an imminent threat to HTX recipients. We investigated the post-transplant effects of early pacemaker dependency immediately after HTX in a large cohort of HTX recipients, focusing on mortality, causes of death, and the requirement for PPM implantation after HTX. A total of 127 of 699 HTX recipients (18.2%) had early pacemaker dependency after HTX, including 52 patients with sinoatrial conduction disturbances (40.9%) and 75 patients with atrioventricular conduction disturbances (59.1%). We found a significantly higher 1-year overall mortality after HTX (55.9% vs. 15.2%, *p* < 0.001) in patients with early pacemaker dependency after HTX along with a significantly higher percentage of death due to graft failure within 1 year after HTX (25.2% vs. 4.2%, *p* < 0.001). Our multivariate analysis showed that early pacemaker dependency after HTX is an independent risk factor for 1-year mortality after HTX (HR: 5.226, 95% CI: 3.738–7.304, *p* < 0.001). Furthermore, patients with early pacemaker dependency after HTX showed a significantly higher rate of 30-day (7.1% vs. 0.4%, *p* < 0.001) and 1-year (11.8% vs. 0.5%, *p* < 0.001) PPM implantation after HTX than patients without early pacemaker dependency after HTX.

In summary, based upon our findings, patients with early pacemaker dependency after HTX had a significantly higher rate of post-transplant mortality, graft failure, and permanent pacemaker implantation, indicating that early pacemaker dependency after HTX is a strong marker of early graft failure.

## Figures and Tables

**Table 1:** Demographics—stratified by early pacemaker dependency after HTX.

Parameter	All Patients (*n* = 699)	Early Pacemaker Dependency (*n* = 127)	No Early Pacemaker Dependency (*n* = 572)	Difference	95% CI	*p*-Value	
Recipient data							
Age (years), mean ± SD	52.0 ± 10.4	53.0 ± 10.8	51.8 ± 10.3	1.2	−0.9–3.3	0.249	
Male sex, *n* (%)	542 (77.5%)	94 (74.0%)	448 (78.3%)	4.3%	−4.0–12.6%	0.293	
BMI (kg/m^2^), mean ± SD	25.1 ± 4.0	25.3 ± 4.0	25.0 ± 4.0	0.3	−0.5–1.1	0.494	
Arterial hypertension, *n* (%)	380 (54.4%)	70 (55.1%)	310 (54.2%)	0.9%	−8.7–10.5%	0.850	
Dyslipidemia, *n* (%)	441 (63.1%)	82 (64.6%)	359 (62.8%)	1.8%	−7.4–11.0%	0.703	
Diabetes mellitus, *n* (%)	225 (32.2%)	43 (33.9%)	182 (31.8%)	2.1%	−7.0–11.2%	0.656	
Peripheral artery disease, *n* (%)	85 (12.2%)	11 (8.7%)	74 (12.9%)	4.2%	−1.4–9.8%	0.182	
COPD, *n* (%)	162 (23.2%)	35 (27.6%)	127 (22.2%)	5.4%	−3.1–13.9%	0.196	
History of smoking, *n* (%)	419 (59.9%)	74 (58.3%)	345 (60.3%)	2.0%	−7.5–11.5%	0.670	
Chronic kidney disease ^, *n* (%)	398 (56.9%)	85 (66.9%)	313 (54.7%)	12.2%	3.1–21.3%	0.012	*
eGFR (mL/min/1.73 m^2^), mean ± SD	60.4 ± 21.9	55.9 ± 20.8	61.4 ± 22.0	5.5	1.5–9.5	0.008	*
Previous open-heart surgery							
Overall open-heart surgery, *n* (%)	224 (32.0%)	38 (29.9%)	186 (32.5%)	2.6%	−6.2–11.4%	0.571	
CABG surgery, *n* (%)	84 (12.0%)	14 (11.0%)	70 (12.2%)	1.2%	−4.9–7.3%	0.703	
Other surgery °, *n* (%)	77 (11.0%)	11 (8.7%)	66 (11.5%)	2.8%	−2.8–8.4%	0.349	
VAD surgery, *n* (%)	86 (12.3%)	17 (13.4%)	69 (12.1%)	1.3%	−5.2–7.8%	0.681	
Principal diagnosis for HTX							
Ischemic CMP, *n* (%)	221 (31.6%)	44 (34.7%)	177 (31.0%)	3.7%	−5.4–12.8%	0.417	
Non-ischemic CMP, *n* (%)	375 (53.6%)	69 (54.3%)	306 (53.5%)	0.8%	−8.8–10.4%	0.865	
Valvular heart disease, *n* (%)	36 (5.2%)	6 (4.7%)	30 (5.2%)	0.5%	−3.6–4.6%	0.810	
Cardiac amyloidosis, *n* (%)	67 (9.6%)	8 (6.3%)	59 (10.3%)	4.0%	−0.9–8.9%	0.164	
Donor data							
Age (years), mean ± SD	41.7 ± 13.5	45.0 ± 13.0	40.9 ± 13.6	4.1	1.6–6.6	0.002	*
Male sex, *n* (%)	307 (43.9%)	55 (43.3%)	252 (44.1%)	0.8%	−8.7–10.3%	0.878	
BMI (kg/m^2^), mean ± SD	25.1 ± 4.3	25.6 ± 4.6	24.9 ± 4.2	0.7	−0.2–1.6	0.103	
Transplant sex mismatch							
Mismatch, *n* (%)	302 (43.2%)	57 (44.9%)	245 (42.8%)	2.1%	−7.5–11.7%	0.673	
Donor (m) to recipient (f), *n* (%)	33 (4.7%)	9 (7.1%)	24 (4.2%)	2.9%	−1.9–7.7%	0.165	
Donor (f) to recipient (m), *n* (%)	269 (38.5%)	48 (37.8%)	221 (38.6%)	0.8%	−8.5–10.1%	0.860	
Perioperative data							
Biatrial anastomosis, *n* (%)	165 (23.6%)	45 (35.4%)	120 (21.0%)	14.4%	5.4–23.4%	0.001	*
*Bicaval anastomosis, *n* (%)	534 (76.4%)	82 (64.6%)	452 (79.0%)	14.4%	5.4–23.4%	0.001	*
Total ischemic time (min), mean ± SD	224.7 ± 67.2	223.6 ± 71.1	225.0 ± 66.3	1.4	−12.1–14.9	0.838	
Cold ischemic time (min), mean ± SD	142.1 ± 59.5	143.3 ± 60.9	141.8 ± 59.2	1.5	−10.2–13.2	0.806	
Warm ischemic time (min), mean ± SD	82.7 ± 27.4	80.3 ± 24.8	83.2 ± 28.0	2.9	−2.0–7.8	0.249	
CPB time (min), mean ± SD	177.4 ± 68.1	185.5 ± 79.7	175.6 ± 65.2	9.9	−5.0–24.8	0.195	
Duration of surgery (min), mean ± SD	329.2 ± 121.3	336.7 ± 129.6	327.5 ± 119.5	9.2	−15.4–33.8	0.467	

BMI = body mass index; CABG = coronary artery bypass graft; CI = confidence interval; CMP = cardiomyopathy; COPD = chronic obstructive pulmonary disease; CPB = cardiopulmonary bypass; f = female; eGFR = estimated glomerular filtration rate; HTX = heart transplantation; m = male; *n* = number; SD = standard deviation; VAD = ventricular assist device; ^ = eGFR < 60 mL/min/1.73 m^2^; ° = congenital, valvular, or ventricular surgery; * = statistically significant (*p* < 0.050).

**Table 2:** Demographics—stratified by early conduction disturbance after HTX.

Parameter	Early Pacemaker Dependency (*n* = 127)	Sinoatrial Conduction Disturbance (*n* = 52)	Atrioventricular Conduction Disturbance (*n* = 75)	Difference	95% CI	*p*-Value	
Recipient data							
Age (years), mean ± SD	53.0 ± 10.8	53.5 ± 9.4	52.6 ± 11.7	0.9	−2.8–4.6	0.633	
Male sex, *n* (%)	94 (74.0%)	40 (76.9%)	54 (72.0%)	4.9%	−10.4–20.2%	0.534	
BMI (kg/m^2^), mean ± SD	25.3 ± 4.0	25.6 ± 4.2	25.1 ± 3.8	0.5	−0.9–1.9	0.493	
Arterial hypertension, *n* (%)	70 (55.1%)	29 (55.8%)	41 (54.7%)	1.1%	−16.5–18.7%	0.902	
Dyslipidemia, *n* (%)	82 (64.6%)	33 (63.5%)	49 (65.3%)	1.8%	−15.2–18.8%	0.828	
Diabetes mellitus, *n* (%)	43 (33.9%)	17 (32.7%)	26 (34.7%)	2.0%	−14.7–18.7%	0.817	
Peripheral artery disease, *n* (%)	11 (8.7%)	4 (7.7%)	7 (9.3%)	1.6%	−8.2–11.4%	0.746	
COPD, *n* (%)	35 (27.6%)	14 (26.9%)	21 (28.0%)	1.1%	−14.7–16.9%	0.894	
History of smoking, *n* (%)	74 (58.3%)	29 (55.8%)	45 (60.0%)	4.2%	−13.3–21.7%	0.634	
Chronic kidney disease ^, *n* (%)	85 (66.9%)	34 (65.4%)	51 (68.0%)	2.6%	−14.1–19.3%	0.758	
eGFR (mL/min/1.73 m^2^), mean ± SD	55.9 ± 20.8	56.1 ± 19.8	55.8 ± 21.6	0.3	−7.0–7.6	0.938	
Previous open-heart surgery							
Overall open-heart surgery, *n* (%)	38 (29.9%)	13 (25.0%)	25 (33.3%)	8.3%	−7.6–24.2%	0.313	
CABG surgery, *n* (%)	14 (11.0%)	4 (7.7%)	10 (13.3%)	5.6%	−5.0–16.2%	0.318	
Other surgery °, *n* (%)	11 (8.7%)	2 (3.8%)	9 (12.0%)	8.2%	−0.8–17.2%	0.108	
VAD surgery, *n* (%)	17 (13.4%)	8 (15.4%)	9 (12.0%)	3.4%	−8.9–15.7%	0.582	
Principal diagnosis for HTX							
Ischemic CMP, *n* (%)	44 (34.7%)	17 (32.7%)	27 (36.0%)	3.3%	−13.5–20.1%	0.700	
Non-ischemic CMP, *n* (%)	69 (54.3%)	30 (57.7%)	39 (52.0%)	5.7%	−11.9–23.3%	0.527	
Valvular heart disease, *n* (%)	6 (4.7%)	1 (1.9%)	5 (6.7%)	4.8%	−2.0–11.6%	0.215	
Cardiac amyloidosis, *n* (%)	8 (6.3%)	4 (7.7%)	4 (5.3%)	2.4%	−6.4–11.2%	0.591	
Donor data							
Age (years), mean ± SD	45.0 ± 13.0	45.5 ± 12.1	44.6 ± 13.6	0.9	−3.6–5.4	0.715	
Male sex, *n* (%)	55 (43.3%)	26 (50.0%)	29 (38.7%)	11.3%	−6.2–28.8%	0.205	
BMI (kg/m^2^), mean ± SD	25.6 ± 4.6	25.2 ± 4.3	26.0 ± 4.8	0.8	−0.8–2.4	0.347	
Transplant sex mismatch							
Mismatch, *n* (%)	57 (44.9%)	20 (38.5%)	37 (49.3%)	10.8%	−6.6– 28.2%	0.226	
Donor (m) to recipient (f), *n* (%)	9 (7.1%)	3 (5.8%)	6 (8.0%)	2.2%	−6.6–11.0%	0.630	
Donor (f) to recipient (m), *n* (%)	48 (37.8%)	17 (32.7%)	31 (41.3%)	8.6%	−8.3–25.5%	0.323	
Perioperative data							
Biatrial anastomosis, *n* (%)	45 (35.4%)	31 (59.6%)	14 (18.7%)	40.9%	24.9–56.9%	<0.001	*
Bicaval anastomosis, *n* (%)	82 (64.6%)	21 (40.4%)	61 (81.3%)	40.9%	24.9–56.9%	<0.001	*
Total ischemic time (min), mean ± SD	223.6 ± 71.1	220.2 ± 84.4	225.9 ± 60.7	5.7	−21.0–32.4	0.678	
Cold ischemic time (min), mean ± SD	143.3 ± 60.9	139.7 ± 73.0	145.7 ± 51.2	6.0	−17.0–29.0	0.608	
Warm ischemic time (min), mean ± SD	80.3 ± 24.8	80.5 ± 27.2	80.2 ± 23.2	0.3	−8.8–9.4	0.940	
CPB time (min), mean ± SD	185.5 ± 79.7	181.2 ± 80.6	188.4 ± 79.5	7.2	−21.1–35.5	0.616	
Duration of surgery (min), mean ± SD	336.7 ± 129.6	326.3 ± 129.8	343.8 ± 129.9	17.5	−28.4–63.4	0.457	

BMI = body mass index; CABG = coronary artery bypass graft; CI = confidence interval; CMP = cardiomyopathy; COPD = chronic obstructive pulmonary disease; CPB = cardiopulmonary bypass; f = female; eGFR = estimated glomerular filtration rate; HTX = heart transplantation; m = male; *n* = number; SD = standard deviation; VAD = ventricular assist device; ^ = eGFR < 60 ml/min/1.73 m^2^; ° = congenital, valvular, or ventricular surgery; * = statistically significant (*p* < 0.050).

**Table 3:** Medications—stratified by early pacemaker dependency after HTX.

Parameter	All Patients (*n* = 699)	Early Pacemaker Dependency (*n* = 127)	No Early Pacemaker Dependency (*n* = 572)	Difference	95% CI	*p*-Value
Immunosuppressive drug therapy						
Cyclosporine A, *n* (%)	349 (49.9%)	68 (53.5%)	281 (49.1%)	4.4%	−5.2–14.0%	0.368
Tacrolimus, *n* (%)	350 (50.1%)	59 (46.5%)	291 (50.9%)	4.4%	−5.2–14.0%	0.368
Azathioprine, *n* (%)	267 (38.2%)	57 (44.9%)	210 (36.7%)	8.2%	−1.3–17.7%	0.087
Mycophenolic acid, *n* (%)	432 (61.8%)	70 (55.1%)	362 (63.3%)	8.2%	−1.3–17.7%	0.087
Steroids, *n* (%)	699 (100.0%)	127 (100.0%)	572 (100.0%)	0.0%	n. a.	n. a.
Concomitant medications						
ASA, *n* (%)	104 (14.9%)	16 (12.6%)	88 (15.4%)	2.8%	−3.7–9.3%	0.425
Beta blocker, *n* (%)	130 (18.6%)	21 (16.5%)	109 (19.1%)	2.6%	−4.6–9.8%	0.509
Ivabradine, *n* (%)	83 (11.9%)	14 (11.0%)	69 (12.1%)	1.1%	−5.0–7.2%	0.743
Calcium channel blocker, *n* (%)	185 (26.5%)	30 (23.6%)	155 (27.1%)	3.5%	−4.7–11.7%	0.422
ACE inhibitor/ARB, *n* (%)	303 (43.3%)	46 (36.2%)	257 (44.9%)	8.7%	−0.6–18.0%	0.073
Diuretic, *n* (%)	699 (100.0%)	127 (100.0%)	572 (100.0%)	0.0%	n. a.	n. a.
Statin, *n* (%)	314 (44.9%)	49 (38.6%)	265 (46.3%)	7.7%	−1.7–17.1%	0.112
Gastric protection drug ^†^, *n* (%)	699 (100.0%)	127 (100.0%)	572 (100.0%)	0.0%	n. a.	n. a.

ACE inhibitor = angiotensin-converting-enzyme inhibitor; ARB = angiotensin II receptor blocker; ASA = acetylsalicylic acid; CI = confidence interval; HTX = heart transplantation; *n* = number; n. a. = not applicable; ^†^ = gastric protection drug defined as proton pump inhibitor (PPI) or histamine receptor (H_2_) blocker.

**Table 4:** Medications—stratified by early conduction disturbance after HTX.

Parameter	Early Pacemaker Dependency (*n* = 127)	Sinoatrial Conduction Disturbance (*n* = 52)	Atrioventricular Conduction Disturbance (*n* = 75)	Difference	95% CI	*p*-Value
Immunosuppressive drug therapy						
Cyclosporine A, *n* (%)	68 (53.5%)	28 (53.8%)	40 (53.3%)	0.5%	−17.1–18.1%	0.955
Tacrolimus, *n* (%)	59 (46.5%)	24 (46.2%)	35 (46.7%)	0.5%	−17.1–18.1%	0.955
Azathioprine, *n* (%)	57 (44.9%)	26 (50.0%)	31 (41.3%)	8.7%	−8.9–26.3%	0.334
Mycophenolic acid, *n* (%)	70 (55.1%)	26 (50.0%)	44 (58.7%)	8.7%	−8.9–26.3%	0.334
Steroids, *n* (%)	127 (100.0%)	52 (100.0%)	75 (100.0%)	0.0%	n. a.	n. a.
Concomitant medications						
ASA, *n* (%)	16 (12.6%)	7 (13.5%)	9 (12.0%)	1.5%	−10.3–13.3%	0.807
Beta blocker, *n* (%)	21 (16.5%)	6 (11.5%)	15 (20.0%)	8.5%	−4.0–21.0%	0.207
Ivabradine, *n* (%)	14 (11.0%)	5 (9.6%)	9 (12.0%)	2.4%	−8.5–13.3%	0.673
Calcium channel blocker, *n* (%)	30 (23.6%)	13 (25.0%)	17 (22.7%)	2.3%	−12.8–17.4%	0.761
ACE inhibitor/ARB, *n* (%)	46 (36.2%)	20 (38.5%)	26 (34.7%)	3.8%	−13.3–20.9%	0.662
Diuretic, *n* (%)	127 (100.0%)	52 (100.0%)	75 (100.0%)	0.0%	n. a.	n. a.
Statin, *n* (%)	49 (38.6%)	16 (30.8%)	33 (44.0%)	13.2%	−3.6–30.0%	0.132
Gastric protection drug ^†^, *n* (%)	127 (100.0%)	52 (100.0%)	75 (100.0%)	0.0%	n. a.	n. a.

ACE inhibitor = angiotensin-converting-enzyme inhibitor; ARB = angiotensin II receptor blocker; ASA = acetylsalicylic acid; CI = confidence interval; HTX = heart transplantation; *n* = number; n. a. = not applicable; ^†^ = gastric protection drug defined as proton pump inhibitor (PPI) or histamine receptor (H_2_) blocker.

**Table 5:** Causes of death within 30 days after HTX. (**a**) Stratified by early pacemaker dependency after HTX. (**b**) Stratified by early conduction disturbance after HTX.

**(a)**							
**Parameter**	**All Patients** **(*n* = 699)**	**Early** **Pacemaker** **Dependency** **(*n* = 127)**	**No Early** **Pacemaker** **Dependency** **(*n* = 572)**	**Difference**	**95% CI**	***p*-Value**	
Graft failure, *n* (%)	43 (6.2%)	29 (22.8%)	14 (2.4%)	20.4%	13.0–27.8%	<0.001	*
Acute rejection, *n* (%)	3 (0.4%)	2 (1.6%)	1 (0.2%)	1.4%	0.2–2.6%	0.029	*
Infection/sepsis, *n* (%)	14 (2.0%)	5 (3.9%)	9 (1.6%)	2.3%	−1.2–5.8%	0.085	
Malignancy, *n* (%)	0 (0.0%)	0 (0.0%)	0 (0.0%)	0.0%	n. a.	n. a.	
Thromboembolic event/bleeding, *n* (%)	8 (1.1%)	4 (3.2%)	4 (0.7%)	2.5%	0.6–5.6%	0.019	*
All causes, *n* (%)	68 (9.7%)	40 (31.5%)	28 (4.9%)	26.6%	18.3–34.9%	<0.001	*
**(b)**							
**Parameter**	**Early** **Pacemaker** **Dependency** **(*n* = 127)**	**Sinoatrial** **Conduction Disturbance** **(*n* = 52)**	**Atrioventricular** **Conduction Disturbance** **(*n* = 75)**	**Difference**	**95% CI**	***p*-Value**	
Graft failure, *n* (%)	29 (22.8%)	10 (19.3%)	19 (25.4%)	6.1%	−8.5–20.7%	0.420	
Acute rejection, *n* (%)	2 (1.6%)	1 (1.9%)	1 (1.3%)	0.6%	−3.9–5.1%	0.793	
Infection/sepsis, *n* (%)	5 (3.9%)	1 (1.9%)	4 (5.3%)	3.4%	−2.9–9.7%	0.331	
Malignancy, *n* (%)	0 (0.0%)	0 (0.0%)	0 (0.0%)	0.0%	n. a.	n. a.	
Thromboembolic event/bleeding, *n* (%)	4 (3.2%)	1 (1.9%)	3 (4.0%)	2.1%	−3.7–7.9%	0.510	
All causes, *n* (%)	40 (31.5%)	13 (25.0%)	27 (36.0%)	11.0%	−5.0–27.0%	0.189	

CI = confidence interval; HTX = heart transplantation; *n* = number; n. a. = not applicable; * = statistically significant (*p* < 0.050).

**Table 6:** Causes of death within 1 year after HTX. (**a**) Stratified by early pacemaker dependency after HTX. (**b**) Stratified by early conduction disturbance after HTX.

**(a)**							
**Parameter**	**All Patients** **(*n* = 699)**	**Early** **Pacemaker** **Dependency** **(*n* = 127)**	**No early** **Pacemaker** **Dependency** **(*n* = 572)**	**Difference**	**95% CI**	***p*-Value**	
Graft failure, *n* (%)	56 (8.0%)	32 (25.2%)	24 (4.2%)	21.0%	13.3–28.7%	<0.001	*
Acute rejection, *n* (%)	5 (0.7%)	2 (1.6%)	3 (0.5%)	1.1%	−1.2–3.4%	0.204	
Infection/sepsis, *n* (%)	80 (11.5%)	29 (22.8%)	51 (8.9%)	13.9%	6.2–21.6%	<0.001	*
Malignancy, *n* (%)	3 (0.4%)	1 (0.8%)	2 (0.4%)	0.4%	−1.1–2.1%	0.495	
Thromboembolic event/bleeding, *n* (%)	14 (2.0%)	7 (5.5%)	7 (1.2%)	4.3%	0.2–8.4%	0.002	*
All causes, *n* (%)	158 (22.6%)	71 (55.9%)	87 (15.2%)	40.7%	31.6–49.8%	<0.001	*
**(b)**							
**Parameter**	**Early** **Pacemaker** **Dependency** **(*n* = 127)**	**Sinoatrial** **Conduction Disturbance** **(*n* = 52)**	**Atrioventricular** **Conduction** **Disturbance** **(*n* = 75)**	**Difference**	**95% CI**	***p*-Value**	
Graft failure, *n* (%)	32 (25.2%)	13 (25.0%)	19 (25.3%)	0.3%	−15.1–15.7%	0.966	
Acute rejection, *n* (%)	2 (1.6%)	1 (1.9%)	1 (1.3%)	0.6%	−4.0–5.2%	0.793	
Infection/sepsis, *n* (%)	29 (22.8%)	12 (23.1%)	17 (22.7%)	0.4%	−14.5–15.3%	0.957	
Malignancy, *n* (%)	1 (0.8%)	0 (0.0%)	1 (1.3%)	1.3%	−1.3–3.9%	0.403	
Thromboembolic event/bleeding, *n* (%)	7 (5.5%)	2 (3.8%)	5 (6.7%)	2.9%	−4.8–10.6%	0.493	
All causes, *n* (%)	71 (55.9%)	28 (53.8%)	43 (57.3%)	3.5%	−14.1–21.1%	0.697	

CI = confidence interval; HTX = heart transplantation; *n* = number; * = statistically significant (*p* < 0.050).

**Table 7:** Multivariate analysis for 1-year mortality after HTX.

Parameter	Hazard Ratio	95% CI	*p*-Value	
Recipient age (years)	1.004	0.987–1.021	0.670	
Recipient BMI (kg/m^2^)	0.982	0.941–1.025	0.411	
Recipient eGFR (mL/min/1.73 m^2^)	0.992	0.984–1.001	0.062	
Donor age (years)	1.005	0.992–1.018	0.469	
Donor BMI (kg/m^2^)	0.961	0.922–1.002	0.063	
Biatrial anastomosis (in total)	0.775	0.505–1.188	0.242	
Total ischemic time (min)	1.000	0.997–1.003	0.975	
Early pacemaker dependency after HTX (in total)	5.226	3.738–7.304	<0.001	*

BMI = body mass index; CI = confidence interval; eGFR = estimated glomerular filtration rate; HTX = heart transplantation; * = statistically significant (*p* < 0.050).

**Table 8:** Permanent pacemaker implantation after HTX. (**a**) Stratified by early pacemaker dependency after HTX. (**b**) Stratified by early conduction disturbance after HTX.

**(a)**							
**Parameter**	**All Patients** **(*n* = 699)**	**Early** **Pacemaker** **Dependency** **(*n* = 127)**	**No Early** **Pacemaker** **Dependency** **(*n* = 572)**	**Difference**	**95% CI**	***p*-Value**	
PPM within 30 days after HTX, *n* (%)	11 (1.6%)	9 (7.1%)	2 (0.4%)	6.7%	2.2–11.2%	<0.001	*
PPM within 1 year after HTX, *n* (%)	18 (2.6%)	15 (11.8%)	3 (0.5%)	11.3%	5.7–16.9%	<0.001	*
**(b)**							
**Parameter**	**Early** **Pacemaker** **Dependency** **(*n* = 127)**	**Sinoatrial** **Conduction Disturbance** **(*n* = 52)**	**Atrioventricular** **Conduction Disturbance** **(*n* = 75)**	**Difference**	**95% CI**	***p*-Value**	
PPM within 30 days after HTX, *n* (%)	9 (7.1%)	3 (5.8%)	6 (8.0%)	2.2%	−6.6–11.0%	0.630	
PPM within 1 year after HTX, *n* (%)	15 (11.8%)	8 (15.4%)	7 (9.3%)	6.1%	−5.7–17.9%	0.299	

CI = confidence interval; HTX = heart transplantation; *n* = number; PPM = permanent pacemaker; * = statistically significant (*p* < 0.050).

**Table 9:** Results for 1-year mortality after HTX in patients with early pacemaker dependency after HTX stratified by PPM within 1 year after HTX.

Parameter	Early Pacemaker Dependency (*n* = 127)	PPM Within 1 Year After HTX (*n* = 15)	No PPM Within 1 Year After HTX (*n* = 112)	Difference	95% CI	*p*-Value	
Mortality ≤ 30 days after HTX, *n* (%)	40 of 127 (31.5%)	0 of 15 (0.0%)	40 of 112 (35.7%)	35.7%	26.8–44.6%	0.005	*
Mortality 31–365 days after HTX, *n* (%)	31 of 87 (35.6%)	8 of 15 (53.3%)	23 of 72 (31.9%)	21.4%	−6.1–48.9%	0.116	
Mortality ≤ 365 days after HTX, *n* (%)	71 of 127 (55.9%)	8 of 15 (53.3%)	63 of 112 (56.3%)	3.0%	−23.9–29.9%	0.831	

CI = confidence interval; HTX = heart transplantation; PPM = permanent pacemaker; *n* = number; * = statistically significant (*p* < 0.050).

**Table 10:** Results for 1-year mortality after HTX stratified by HTX era.

Parameter	All Patients (*n* = 699)	Early Pacemaker Dependency (*n* = 127)	No Early Pacemaker Dependency (*n* = 572)	Difference	95% CI	*p*-Value	
HTX era 1989–1996, *n* (%)	33 (4.7%)	23 (18.1%)	10 (1.7%)	16.4%	9.6–23.2%	<0.001	*
HTX era 1997–2004, *n* (%)	35 (5.0%)	17 (13.4%)	18 (3.2%)	10.2%	4.1–16.3%	<0.001	*
HTX era 2005–2012, *n* (%)	47 (6.7%)	15 (11.8%)	32 (5.6%)	6.2%	0.3–12.1%	0.011	*
HTX era 2013–2022, *n* (%)	43 (6.2%)	16 (12.6%)	27 (4.7%)	7.9%	1.9–13.9%	<0.001	*
Overall HTX period 1989–2022, *n* (%)	158 (22.6%)	71 (55.9%)	87 (15.2%)	40.7%	31.6–49.8%	<0.001	*

CI = confidence interval; HTX = heart transplantation; *n* = number; * = statistically significant (*p* < 0.050).

## Data Availability

The original contributions presented in this study are included in the article, and further inquiries can be directed to the corresponding author.
